# Development and validation of the autotransplanted maxillary canine radiological index

**DOI:** 10.1002/cre2.125

**Published:** 2018-08-17

**Authors:** Koenraad Grisar, Jasper Vanpoecke, Margot Raes, Emad Ali Albdour, Guy Willems, Constantinus Politis, Reinhilde Jacobs

**Affiliations:** ^1^ OMFS IMPATH Research Group, Department of Imaging and Pathology, Faculty of Medicine, University of Leuven and Department of Oral and Maxillofacial Surgery University Hospitals Leuven Belgium; ^2^ Department of Oral Health Sciences University of Leuven, University Hospitals Leuven Belgium; ^3^ Department of Dental Medicine Karolinska Institutet Sweden

**Keywords:** autotransplantation, canine, cuspid, impacted, maxillary

## Abstract

The purpose of this study was to propose and validate an index evaluating 2D and 3D radiographic variables of autotransplanted maxillary canines. Setting and sample population are from the Department of Oral and Maxillofacial Surgery at University Hospitals Leuven. Eight oral‐maxillofacial surgeons rated 12 autotransplanted maxillary canines and adjacent bone using 11 rating variables. A new autotransplanted maxillary canine radiological index (AMCRI) was proposed. It consisted of 11 variables. These variables were based on 2D (intraoral) and 3D Cone Beam Computed Tomography (CBCT) radiographs. Intraclass correlation coefficient (ICC) and Fleiss's kappa statistics were performed to analyze intrarater and interrater agreement. Considering cumulative assessment of the AMCRI, the mean ICC value for the interrater agreement of the eight examiners was 0.94, representing an excellent agreement. Intrarater agreement was 0.91. The AMCRI is an objective tool in rating radiological outcome of autotransplanted canines and adjacent bone, when compared with the contralateral canine.

## 1 INTRODUCTION

Maxillary canine impaction has been reported to occur in 2–3% of the population (Bedoya & Park, 2009). Autotransplantation is a potential treatment option in cases in which surgical exposure and orthodontic traction are not successful or impossible (Arikan, Nizam, & Sonmez, 2008; Grisar et al., 2018). This treatment could be preferred considering an unfavorable displacement, as well as failure of orthodontic alignment due to immobility or because the patient refused a conventional orthodontic therapy (Ericson & Kurol, 2000).

Ideally, an autotransplanted tooth can be present in the jaw bone for the patient's entire life. However, there are other reasons supporting this treatment, even if life‐long survival cannot be achieved. Transplanted teeth have the capacity to preserve the alveolar ridge, especially during growth, during which dental implants are contraindicated (Andersson et al., 2012; Czochrowska, Stenvik, Bjercke, & Zachrisson, 2002; Schwartz‐Arad, Levin, & Ashkenazi, 2004). By analogy, avulsed teeth, even those with poor prognoses, are recommended for replantation in cases of dental trauma (Andersson et al., 2012).

An important part of the follow‐up of an autotransplanted maxillary canine is the radiographic control with intraoral and 3D CBCT images. A standardized radiological evaluation protocol is not yet existing. It was our aim to develop a brief, simple, and easy‐to‐use questionnaire to objectively score the radiological appearance of autotransplanted maxillary canines in the long‐term follow‐up. This index can be helpful for the general dentist, orthodontist, and maxillofacial surgeon. It can be used in the screening for important variables determining outcome and the assessment of the final result.

The aim of the present report is to introduce the autotransplanted maxillary canine radiological index (AMCRI), based on a combined 2D and 3D radiological evaluation and validated in a random sample of autotransplanted maxillary canines (Andreasen & Hjørting‐Hansen, 1966; Huth et al., 2013; Sugai et al., 2010).

## 2 MATERIAL AND METHODS

This study was conducted at the Departement of Oral and Maxillofacial Surgery, University Hospital Leuven, Belgium. The study protocol was approved by the Ethics Committee of the University Hospital Leuven, Belgium (s number: s53225).

Eleven radiological variables were selected based on the available evidence in literature regarding their relation to treatment outcome (Almpani, Papageorgiou, & Papadopoulos, 2015; Atala‐Acevedo et al., 2017; Chung, Tu, Lin, & Lu, 2014; Machado, do Nascimento, Ferreira, Mattos, & Vilella, 2016). The radiological variables were based on the follow‐up protocols of multiple studies concerning autotransplantation of maxillary canines (Ahlberg, Bystedt, Eliasson, & Odenrick, 1983; Arikan et al., 2008; Chambers, Reade, & Poker, 1988; Gonnissen et al., 2010; Hall & Reade, 1983; Kallu, Vinckier, Politis, Mwalili, & Willems, 2005; Kvint, Lindsten, Magnusson, Nilsson, & Bjerklin, 2010; Lownie, Cleaton‐Jones, Fatti, & Lownie, 1986; Patel, Fanshawe, Bister, & Cobourne, 2011; Pogrel, 1987; Sagne, Lennartsson, & Thilander, 1986; Sagne & Thilander, 1997; Schatz & Joho, 1994). Six radiological variables were evaluated both in 2D and 3D imaging, thus having a final 17 variables. All variables and their assessment were described in Tables 1 and 2.

**Table 1:** Autotransplanted maxillary canine radiological index scoring sheet

Parameter	Absent	Present but incomplete	Present
2D radiographic scoring			
Periodontal ligament	2	1	0
Lamina dura	2	1	0
Apical root closure	2	1	0
	**Present**		**Absent**
Apical radioluceny	10		0
Ankylosis	2		0
Root resorption	5		0

3D radiographic scoring			
Periodontal ligament	2	1	0
Lamina dura	2	1	0
Apical root closure	2	1	0
Peritransplant bone volume	2	1	0
	**Present**		**Absent**
Apical radiolucency	10		0
Ankylosis	2		0
Root resorption	5		0
Internal root resorption	5		0
	**Major discrepancy**	**Minor discrepancy**	**No discrepancy**
Vestibular bone height	2	1	0
Vestibular bone thickness	2	1	0
Vestibular prominence canine	2	1	0
Total score	0–5 points = excellent 6–13 points = good 14–20 points = moderate 21 or more points = poor outcome		

**Table 2:** Autotransplanted maxillary canine radiological index variables

Variables	Description	Judgment instructions	Outcome	Figures
Periodontal ligament (PDL)	PDL should be visible on 2D and 3D (no radiological sign of ankyloses)	Judgment made on a 3‐point rating scale	(a) Absent (b) Present but incomplete (c) Present	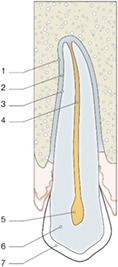 1: lamina dura; 2: periodontal ligament; 3: cementum; 4: pulp canal; 5: pulp chamber; 6: dentin; 7: enamel
Lamina dura	Lamina Dura should be visible on 2D and 3D (no radiological sign of ankyloses)			
Apical root closure	Root closure should be visible on 2D and 3D a result of further development of the autotransplanted tooth.			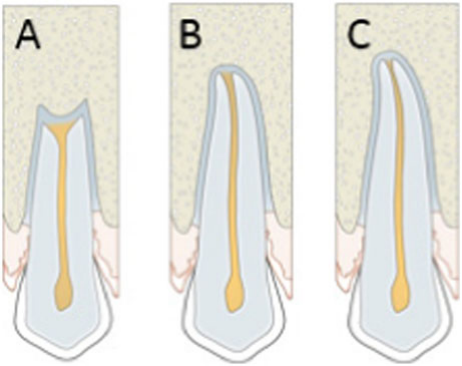 A: open root; B: partially closed root; C: closed root
Peritransplant bone volume	Peritransplant bone volume should be visible (only in 3D) demonstrating further development of surrounding bone.			
Apical radiolucency	Associated with apical infection and poor prognosis	Judgment made on a two‐point rating scale	(a) Absent (b) Present	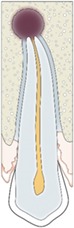
Ankylosis	Disappearance of the PDL space and lamina dura, bone replacement of the root dentin, but no adjacent radiolucency.			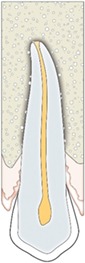
Root resorption	Associated with poor prognosis and radiologically visible as radiolucency on the external root surface of dentin and adjacent bone.			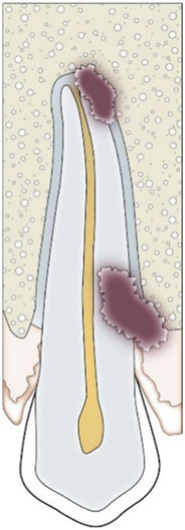
Internal root resorption	Associated with poor prognosis. Only visible on 3D images, presenting as a uniform, circular radiolucent area within pulpal canal.			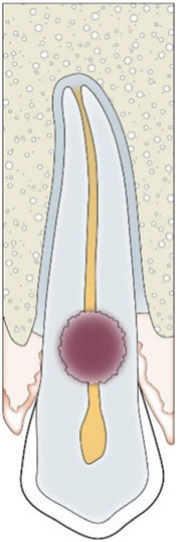
Vestibular bone height	Vestibular bone height (long arrow) can be visible only in 3D imaging as a result of further development of surrounding bone	Judgment on a three‐point rating scale	(a) No discrepancy (b) Minor discrepancy (c) Major discrepancy	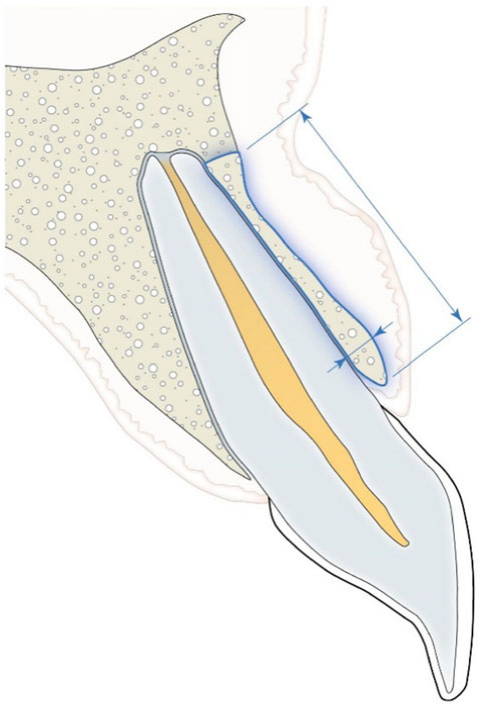
Vestibular bone thickness	Vestibular bone thickness (short arrow) can be visible only in 3D imaging as a result of further development of surrounding bone			
Vestibular prominence canine	Visible only in 3D imaging as a combined result of initial positioning of autotransplanted canine and final orthodontic movements			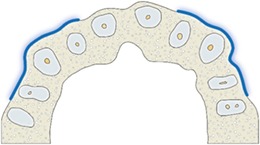

The index comprised the cumulative scoring of the variables. Teeth were evaluated on each of the variables indicated. If indicated, the examined tooth was compared with the contralateral canine tooth. Points were given to each of these items: 0 points for the desired situations; 1 point for a moderate result; and 2, 5, or 10 points for a gross deviation. For the gross deviations, 5 or 10 points were assigned for the variables that were considered to be the most important for the final outcome; 2 points were assigned when the variable was considered to be less important.

It can be noticed that an apical radiolucency suggesting infection, root resorption, or internal root resorption automatically leads to a poor radiological result and can never be accepted as moderate or satisfactory. It should be recognized that patients who had treatment for bilateral impacted maxillary canines are more difficult to assess with the AMCRI.

Before objectively scoring the teeth, the observers were asked to subjectively score each case with “excellent,” “good,” “acceptable,” and “poor” final outcome. These scorings were correlated with the total objective scores. An expert consensus allowed for benchmarking of the rating scale and calibrated scoring with the new index. To test the reliability of the newly developed index, intraobserver and interobserver agreement must be calculated (Landis & Koch, 1977). Nine patients with 12 autotransplanted maxillary canines (five males, four females; mean age 24.3 years) were randomly selected out of the patients database of the Department of Oral and Maxillofacial Surgery, University Hospitals Leuven. Mean follow‐up time was 2.3 years. Minimal follow‐up after autotransplantation was 2 years. Radiological imaging (intra‐oral and CBCT) was collected and standardized (single‐view intraoral radiographs and examiners were provided sections from the CBCT). Observations were performed on standard screens. Initial training and calibrations of all observers were performed. Observations were performed at T0 (baseline), T1 (2 weeks after T0), and T2 (4 weeks after T0) after randomization. Eight examiners (all oral‐maxillofacial surgeons) underwent familiarization with the index, followed by calibration. Each of the transplanted maxillary canines was rated on a form with the 17 items of the rating index. The rating was carried out 3 times by each of the examiners. There was a 2‐week time‐interval period between the ratings to prevent recollection of the first rating. Intraclass correlation coefficient and Fleiss's kappa tests have been calculated to express the intraobserver and interobserver agreement.

## 3 RESULTS

**Figure 1:**
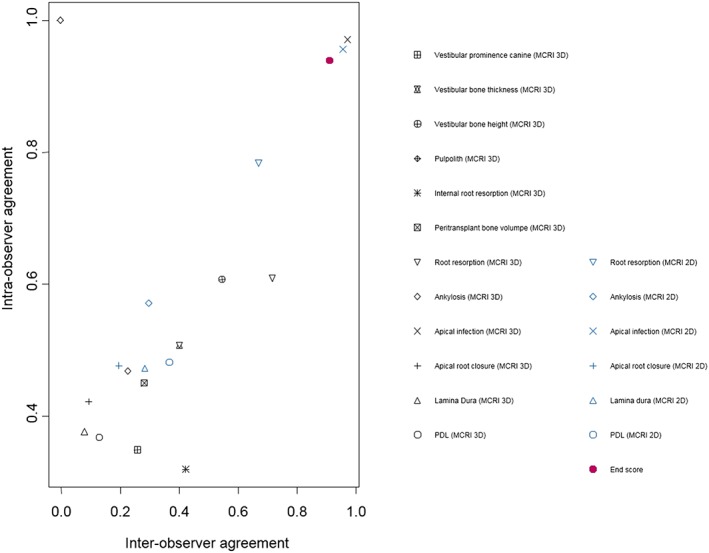
Interobserver and intraobserver agreement (intraclass correlation coefficient and Fleiss's kappa tests). MCRI: maxillary canine radiological index; PDL: periodontal ligament

The intraobserver and interobserver agreement for the 17 variables and final score are listed in Figure 1. It can be noticed that the highest interobserver agreement was obtained when assessing 2D and 3D apical infection, 2D and 3D root resorption, and 3D vestibular bone height. Lowest interoberserver agreement was obtained when assessing 3D lamina dura, 3D apical root closure, and 3D pulpolith.

**Figure 2:**
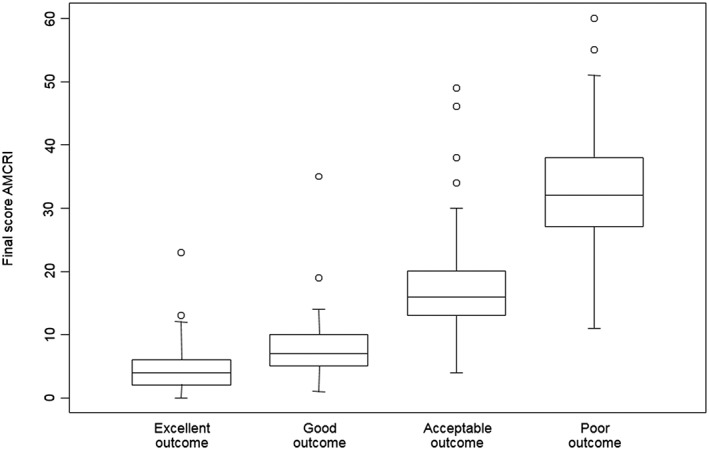
Box plots displaying correlations objective and subjective scoring. *X*‐axis represents the subjective scoring as given by the different observers; *Y*‐axis represents the corresponding mean final objective score on the autotransplanted maxillary canine radiological index (AMCRI). Cutoff values for correlation of objective and subjective scoring were obtained based upon the full range of variation (from min to max), the likely range of variation (the interquartile range), and the median value

The subjective scoring of each observer was correlated with the total scores (Figure 2). Spearman correlation test showed a value of 0.89, demonstrating good correlation.

Based upon these results, the following classification was proposed (Table 3). A total objective score of 0–5 points correlates with an excellent final outcome, a total objective score of 6–13 points with a good final outcome, a total objective score of 14–20 points with an acceptable final outcome, and a total objective score of 21 points or more with a poor final outcome.

**Table 3:** Correlation final score AMCRI with outcome

Total score AMCRI	Final outcome
0–5	Excellent
6–13	Good
14–20	Acceptable
≥21	Poor

*Note*. AMCRI: autotransplanted maxillary canine radiological index.

## 4 DISCUSSION

In the present study, we introduced a new index (AMCRI) and validated it. It was developed considering the lack of a standardized method of evaluating and measuring radiographical outcome after autotransplantation of impacted maxillary canines. The goal was to develop an index that could be used in both research and clinical settings as a guideline for diagnosing and documenting outcome.

High interobserver and intraobserver agreement results on final endscore were obtained (Figure 1). Both 2D and 3D imaging appear to be reliable as tools for assessment of final outcome.

Low scores on interobserver and intraobserver agreement were found when assessing 3D pulpolith. This can be explained by the fact that one observer gave a different score (Arikan et al., 2008) whereas all other observers indicated the same score (0). This creates a major imbalance in Fleiss's kappa statistics, resulting in an interobserver agreement of almost zero.

These initial results with the radiographical index are very promising, but its practical use as a standard procedure has to be confirmed in a large‐scale clinical study.

The index could be a very useful tool in scientific research. Results of the AMCRI might be checked for correlation with the final outcome, whereby a possible correlation and a predictive value can be linked to it. The index could also give a better, objective, insight in one's own results in daily practice.

## 5 CONCLUSIONS

From this study, it can be concluded that the AMCRI is an objective tool in rating radiographical outcome of autotransplanted maxillary canines. Clinicians might find it usefull in daily clinical practice and scientific research. However, one must be aware that this index only judges the radiographical and not the functional outcome of the canine. A poor radiographical result does not imply malfunction, though it can be related to premature loss of the transplanted tooth due to apical infection or root resorption. To verify its clinical applicability, the AMCRI should be used on a larger data sample.

## CONFLICT OF INTEREST

The authors report no conflict of interest related to this study.
